# Helical Gearbox Defect Detection with Machine Learning Using Regular Mesh Components and Sidebands

**DOI:** 10.3390/s24113337

**Published:** 2024-05-23

**Authors:** Iulian Lupea, Mihaiela Lupea, Adrian Coroian

**Affiliations:** 1 Faculty of Industrial Engineering, Robotics and Production Management, Technical University of Cluj-Napoca, 400114 Cluj-Napoca, Romania; adrian.coroian@mep.utcluj.ro; 2 Faculty of Mathematics and Computer Science, Babes-Bolyai University, 400084 Cluj-Napoca, Romania; mihaela.lupea@ubbcluj.ro

**Keywords:** helical gears, regular mesh components, sidebands, triaxial accelerometer sensor, vibration signal, fault detection, machine learning

## Abstract

The current paper presents helical gearbox defect detection models built from raw vibration signals measured using a triaxial accelerometer. Gear faults, such as localized pitting, localized wear on helical pinion tooth flanks, and low lubricant level, are under observation for three rotating velocities of the actuator and three load levels at the speed reducer output. The emphasis is on the strong connection between the gear faults and the fundamental meshing frequency GMF, its harmonics, and the sidebands found in the vibration spectrum as an effect of the amplitude modulation (AM) and phase modulation (PM). Several sets of features representing powers on selected frequency bands or/and associated peak amplitudes from the vibration spectrum, and also, for comparison, time-domain and frequency-domain statistical feature sets, are proposed as predictors in the defect detection task. The best performing detection model, with a testing accuracy of 99.73%, is based on SVM (Support Vector Machine) with a cubic kernel, and the features used are the band powers associated with six GMF harmonics and two sideband pairs for all three accelerometer axes, regardless of the rotation velocities and the load levels.

## 1. Introduction

Finding intelligent methods for gearbox fault detection has become an intensively studied topic in recent decades. This is due to the fact that the early detection of possible faults in gearboxes, and rotating machines, in general, can increase the operational safety of a device, as it can reduce the costs of maintenance and prevent total failure. Within this context, data-driven fault detection models are preferable to the traditional physics-based models, which start simple and progress to more complex mathematically formalized models. Early defect causes include improper lubrication, overheating from extra loads, surrounding bearing failure, the poor design or deficient manufacture of the gear pairs, and water intrusion due to seal failure. The life-limiting element in gear transmission is frequently the fatigue of the materials on contacting surfaces. In this regard, surface treatments such as superfinishing, grinding, or lapping are required on the flank surfaces of the teeth. The condition of the machine changes in terms of temperature, lubrication, noise produced, and vibration profile after a malfunction occurs. Common transducers for machine condition signal acquisition are the accelerometer, microphone, and acoustic emission sensor.

Typical defects on gears include adhesive wear, micro-pitting, pitting, chipping, scuffing, spalling, cracking, tooth breaking, and white structure flaking [1,2]. Most gearbox problems are found to be caused by these. Certain defects, such as micro-pitting and pitting due to Hertzian fatigue, wear, scuffing, and scoring, are linked to issues with lubrication. Non-lubrication-related failures include plastic flow, tooth fractures (due to overloading), and root fillet cracks caused by bending fatigue at the tooth root [2].

It is customary to detect and classify gear faults using the most important statistical features derived from time-domain vibration signals measured on the mechanical structure under observation. Some of the general signal-based statistical parameters are arithmetic mean value (the first moment of the probability density function), mean of absolute values, mean square value, root mean square (rms), variance (the second moment of the probability density function about the mean value), standard deviation (square root from variance), and geometric mean. The properties related to the peaks of the measured signal, such as peak value (the maximum absolute value of the signal), peak to peak, peak to rms (crest factor), impulse factor, and clearance factor, are known as impulsive metrics. Other useful features are the shape factor and zero cross rate [3,4,5]. Higher order moments of the signal include the following ones: moment of the third order, coefficient of skewness (the third moment is normalized using the third power of the standard deviation of the measured signal, resulting in a unitless parameter, which is a measure of the asymmetry of the probability density function about the mean), moment of the fourth order (the fourth moment of the probability density function about the mean), kurtosis (the fourth centralized moment of the signal, normalized by the square of the variance that makes the parameter unitless), and energy operator (normalized kurtosis on the modified original signal) [4,6].

The level of chaos in a system can indicate the emergence or progress of a fault in gears or bearings. The changes in phase space trajectory (associated with the system dynamics) can be identified by observing nonlinear parameters in time-series data, such as the largest Lyapunov exponent, the approximate entropy (which quantifies the amount of regularity or unpredictability in a signal), and the correlation dimension estimation (a measure of the dimensionality of the phase space occupied by the signal) [7].

The Time Synchronous Average (TSA), one of the time-averaging techniques, is a crucial instrument for signal conditioning and gear failure detection (fixed axis gears) since it significantly lowers background noise and periodic events arising from gears other than those tracked or from bearings. Accelerometer and tachometer sensors are needed to average blocks of vibration data (phase-locked periodic segments) representing one revolution of the observed gear. Prior to averaging, the data segments are resampled angle-based. TSA precedes the calculation of gear status (health) condition indicators (CI). A number of statistical time-domain condition indicators, such as ER (energy ratio), energy operator, FM0, FM4, NA4, NB4, M6A, M8A, NA4*, NB4*, M6A*, and M8A*, were derived using TSA regular, difference, or residual signals to detect faults [4,6,8,9,10].

Statistical frequency-domain features help in fault diagnosis through the separation and interpretation of different sources mixed in the vibration signals. The fast Fourier transformation (FFT) is applied to stationary signals, resulting in spectral information. In most of the classification models, derived frequency-domain condition indicators are of great importance. Some of them are statistical, and they are applied to the power spectrum of the signal, resulting in parameters such as the mean frequency of the power spectrum, spectral centroid, spectral spread, spectral skewness, spectral kurtosis, spectral flatness, spectral crest, spectral slope, spectral entropy, Shannon entropy, and so forth [3,11]. Apart from statistical parameters, the spectrum of a gearbox presents important frequencies, such as gear mesh frequencies (GMFs), their harmonics, and sideband pairs associated with the GMFs and harmonics. These are influenced by the shaft’s rotation velocity, gear constructive parameters, and the surface quality of the tooth flanks.

Envelope analysis is particularly useful in detecting and diagnosing gear faults, such as pitting or gear tooth damage, from signals which are periodic, impulsive, and modulated by high-frequency resonance components. The power spectrum of the detected envelope shows the frequency components of the localized faults. Many gear faults are synchronous with the rotation velocity, allowing TSA, while for the bearing fault frequencies (which are not synchronous with shaft velocity), envelope detection is of utmost importance. Among the available techniques, one can mention the Hilbert transform and bandpass rectification [1]. Vibration signals, especially when defects are present, have non-stationary components and may contain several families of periodicities.

Techniques for the fault diagnosis and prognosis of gearboxes under non-stationary operating conditions (along with stationary operating conditions) are available. One can mention the use of spectral kurtosis [12] and the associated fast Kurtogram to find the optimal and narrow frequency band in which the vibrational manifestation (impulsiveness) of the defect (in rolling bearings and gears) is present. Filtering out that band, followed by demodulation and envelope analysis, leads to the determination of the specific frequencies of defects. In [13], a new feature called energy ratio, based on an autoregressive (AR) model, is directly extracted from original non-stationary vibration signals (and not from TSA signals) and used for the diagnosis and prognosis of a gearbox.

Order analysis (particularly order tracking) is an effective method to analyze vibration signals for fault detection on gears and other rotating machine elements under the time-varying rotating shaft [14]. Order analysis helps to identify gear faults that exhibit specific frequency components related to gear meshing, such as gear mesh harmonics, sidebands, and gear mesh frequency modulation. In the spectrum domain, the focus is on orders rather than frequency components. Colormaps from run-up and cost-down and from order tracking can be used in machine learning-based fault diagnosis.

Cepstrum analysis (CA), as a result of the inverse Fourier transform (IFT) of the logarithm of the vibration signal spectrum, is helpful in interpreting the sidebands found in the power spectrum. CA is efficient for signals with many families of periodicities and can be used to detect and quantify families of periodically spaced spectral components such as harmonics, as well as equally spaced modulation sidebands. Cepstrum analysis presents a lack of sensitivity to the position of the accelerometer sensor on the gearbox case, as CA collects (and averages) information about the sidebands from the whole spectrum, reducing the same order of sidebands for various GMF harmonics into a single line in the cepstrum [15].

Classical signal processing methods for nonstationary signals with time-dependent statistics are enumerated in what follows. A good signal interpretation is using spectrograms (the spectrum varies over time) in the combined time–frequency domain presentation [16]. The spectrograms are derived from short-time Fourier transform (STFT) analysis (time and frequency resolution can be controlled by the window length, assuming that the data are piecewise stationary), the Gabor transform (a special case of STFT, the analyzing window being a Gaussian function), the Stockwell transform (ST) [17], and Wigner–Ville distribution (WVD). Spectrograms provide better temporal versus frequency resolution and associated variants, aiming to diminish the interference from cross-terms (CTs) for multi-component signals, smooth pseudo WVD (SPWVD), adaptive directional time–frequency distribution (ADTFD) [18], and WVD enhancement methods such as the one based on generative adversarial networks (WVD-GAN) combined with deep learning techniques [19].

Wavelet transforms (WTs) express the vibration signal in terms of specific functions named wavelets, which are scaled (by compressing or stretching) and shifted in order to reconstruct the observed signal. The vibration signal is decomposed into different time–frequency scales, known as scalograms. Continuous wavelet transform (CWT), discrete wavelet transform (DWT), and wavelet packet transform (WPT, a superior alternative to DWT for time–frequency analysis) are efficient in data analysis for the fault diagnostics of gears and bearings [3,16].

Empirical mode decomposition (EMD), as a time–frequency analysis technique, observes the decomposition of a complicated (measured) signal into a collection of components having embedded simple intrinsic oscillation modes, from high- to low-frequency content, called intrinsic mode functions (IMFs). Further processing can be applied to IMFs, such as computing the instantaneous frequency of every IMF, combining IMFs, applying envelope analysis, and extracting statistical parameters [20]. To enhance the performance of the basic processing methods, hybrid signal processing techniques have also been introduced. The local mean decomposition (LMD) is an iterative and adaptive analysis technique that decomposes the measured signal into a set of functions, each of which is the product of an envelope signal and a purely frequency-modulated (FM) signal [21]. Acoustic emission techniques [22] can be used in conjunction with vibration techniques to monitor gears and bearings.

In the past two decades, intense research has been conducted on tooth gear defect detection from vibration, acoustic, and acoustic emission signals. The studies are based on traditional fault detection methods, such as STFT, CA, WT, WVD, and Hilbert–Huang transform (HHT), followed by processing with machine learning (ML) techniques: SVM (Support Vector Machine), K-NN (K-Nearest-Neighbors), Random Forest, Naïve Bayes, Bayes Net, and NN (Neural Network) [11,21,23,24].

In [25], gearbox fault diagnosis is realized by using a method that combines SVM, wavelet lifting, and rule-based reasoning. The vibration signals are processed with wavelet packet decomposition, and the energy coefficients of each frequency band are calculated and used further as input feature vectors in SVM to predict a normal or faulty state. To distinguish between different faults, the noisy signals are filtered using wavelet lifting while maintaining the machine fault features, and then rule-based reasoning is applied to identify the detailed fault type. Two real validation cases are presented. In one case, the broken tooth is on a helical cylindrical gear, and in the second case, the broken cog belongs to a bevel gear. Gear defects are large, affecting most of the tooth’s length.

To differentiate between the four states (normal, broken, pitting, and wear) of a gearbox, the authors of paper [26] proposed a method based on the SVM model. The input of the SVM is obtained as a fusion of two domain features: time–frequency features and features extracted from the original vibration signals using the 1D-CNN model. The SVM model, optimized by the improved particle swarm optimization (IPSO) algorithm, achieved a very high accuracy of 98.3%.

Paper [27] emphasizes the positive impact of the feature selection process combined with data segmentation techniques (sliding, windowing, and bootstrapping) in diagnosing gearbox faults using machine learning models. An initial set of 13 features in the time domain, extracted from each of the four channels, was subjected to various ranking methods to select the most informative features. In the conducted experiments, several machine learning algorithms were applied to solve the binary classification task (healthy gearbox versus faulty gearbox) in different scenarios, using several selected feature sets and with/without data segmentation. The DT (decision tree), K-NN, SVM, and NN models achieved a significant improvement in accuracy (about 60%), reaching almost 100% when using selected features and appropriate data segmentation.

In [28], the defects correspond to different levels of cracks placed at the tooth base of the gear. The Daubechies44 binary wavelet packet transform, at different wavelet decomposition levels, is applied to the vibration signals, and then statistical features are extracted. Principal component analysis is used for dimensionality reduction of the features. The K-NN classifier, selected from several ML methods, successfully identifies the mentioned faults under different motor speeds and loads.

Deep learning has significantly contributed to automatic fault diagnosis (with very good learning and classification capabilities) by directly extracting, in certain cases from the raw vibration signal, the most relevant features. The main deep learning directions in machine health monitoring systems include Convolutional Neural Networks (1D or 2D CNNs), autoencoders (AEs and variants), Restricted Boltzmann Machines (RBMs and variants), and Recurrent Neural Networks (RNNs) [29,30]. In [31], CNN-based defect detection models use 1D and 2D grayscale images, representing the vibration image of the acquired raw data. In [32], an adaptive stacked CNN with a multi-sensor fusion algorithm fuses the diagnosis output based on vibration signals with the one based on sound signals. In [33], a deep sparse autoencoder network (self-supervised machine learning) is employed for gear pitting detection. Reference [34] utilizes a 1D-CNN trained on raw acoustic emission signals and a Gated Recurrent Unit network trained on raw vibration signals to detect gear pitting faults.

Trendy analysis involves monitoring vibration data over an extended period of time to identify changes in vibration patterns and track the progression of gear faults. An accurate prediction of the gear remaining useful life (RUL) by using a long short-term memory (LSTM) Neural Network with weight amplification can be found in [35].

The current work proposes a method for diagnosing the defect type by observing variations in the vibration signal of the test rig, which are quantified solely in the regular mesh components and associated sidebands. The condition (health) states under observation are poor lubrication, normal state, and two structural defects on a pinion tooth flank, namely, localized pitting and localized wear. Vibrations at the speed reducer case and the rotation velocity at the speed reducer output are measured by using a triaxial accelerometer and a tachometer, respectively. Vibrations are considered for three different motor velocities, three loads for each velocity, and four health states of the reducer. Minor fluctuations in speed and load around the nominal operating conditions are present. Several types of features are extracted using signal processing techniques and then used as predictors in the defect detection task, modeled as a multi-class classification problem.

The aim of the present study is to answer the following research questions:

RQ1: What are the most relevant time-domain and frequency-domain statistical parameters extracted from the vibration signals measured using a triaxial accelerometer (three rotation velocities and three load levels) for detecting the health states of the helical gearbox?

RQ2: To what extent does adding the peak amplitude of the GMF bands on all three axes to the selected statistical features improve the performance of the defect detection task?

RQ3: Accrediting the idea that the defects on gear active tooth flanks are embedded in the amplitude and phase modulation of the vibration signal, how effective is the use of band powers or/and associated peak amplitudes (from the vibration spectrum) on frequency bands associated with GMF, harmonics, and sideband pairs as features for classification?

This paper is structured as follows. Section 2 is dedicated to related studies that emphasize the importance of GMF, harmonics, and their sidebands in capturing the defects on the active tooth flanks. In Section 3, we present the test-rig, the data acquisition, and the data preprocessing, followed by a description of the defects in the input shaft pinion of the speed reducer. The methodology for defect detection, described in Section 4, outlines two approaches, one based on statistical parameters and the other on features extracted from frequency bands of interest in the power spectrum. The experimental results and discussions are presented in Section 5. The last section contains the conclusions and directions for future work.

## 2. Gear Defects, Regular Mesh Components, and Sidebands

The gear faults generate a vibration signal with the spectrum energy distributed over various frequencies. The pattern of energy distribution of the faulty gear makes fault detection possible. Hence, the spectra (generated using FFT) measured for different faults or for a faultless gearbox are discernible; each power spectrum should be unique for a fault size or location, and the power in specific frequency bands is important [5].

Apart from the mentioned general statistical parameters of the vibration signal (in the time domain and frequency domain), important fault classification features take into consideration parameters related to the constitutive machine elements of the rotating machinery under observation. The normal vibrations from a gear pair are mainly produced by the shocks between the teeth as the gears mate during operation. Such a functional parameter is the tooth-meshing rate known as gear mesh frequency (GMF), calculated as the product of $z_{1}$, the number of pinion teeth, and $f_{p}$, the rotational frequency of the pinion shaft. The GMF also equals the product of $z_{2}$, the number of the wheel teeth, and $f_{w}$, the associated/supporting shaft speed (Equation (1)).

(1) $$f_{p}z_{1}=f_{w}z_{2}$$

The GMF, the upper harmonics of the GMF, and their first pair of sidebands are known as regular mesh components. A faulty gear presents on the frequency axis several sideband pairs on both sides of the GMF and of the GMF’s harmonics, at a distance equal to the rotational speed of the faulty gear. Even though the vibration signals measured on the reducer case are distorted by the transmission paths from the source to the transducer, the regular mesh components and the sidebands are important in the spectrum to differentiate faults.

Frequency sidebands are generated by the amplitude modulation (AM) of the GMF component of the measured vibration signal and the frequency modulation (FM) or phase modulation of the same component of the measured signal. When a gear has a local fault, the vibration signal of the gearbox may contain amplitude and phase modulations that are periodic with the rotation frequency ($f_{p}$ or $f_{w}$) of the gear. The modulation of the meshing frequency, as a result of faulty teeth, generates sidebands, which are frequency components equally spaced on both sides of the center frequency [36].

The amplitude modulation of the GMF can be explained by the sensitivity of the vibration amplitude to the tooth loading, based on the variation of the tooth contact pressure. AM and FM under constant rotational speed of the gear pair derive from teeth deflection under transmitted load and teeth geometrical errors. The periodic signal associated with gear mesh frequency is due to deviations from the ideal tooth profile. These deviations are caused by tooth deflection under load and from geometrical errors of the flank profile from gear manufacturing or wear [1]. The deflection of the teeth under load imposes in the vibration signal (in the time domain) a stepped profile as the load is divided between successive pairs of teeth. A couple of tooth pairs are simultaneously engaged in contact at the line of action (which is tangent to both base circles of the gear pair). The deflection variation is reduced for helical gear (versus spur gear) observed in the current speed reducer, where the contact line length is larger in comparison to the spur gear [31]. More tooth pairs are simultaneously in contact in helical gears; therefore, we have a smaller deflection variation on the helical gear while the gear pair meshes. These deflection aspects affect several harmonics of the basic tooth-meshing frequency in the measured vibrational signal. Gear machining errors of the random type are translated into a low spectrum level spread over a large number of harmonics [1]. To alleviate the spectrum amplitude at the GMF, specific geometric deviations from the ideal involute profiles are practiced intentionally by removing metal from the tip of each tooth with a maximum at the tip, and gradually reducing to zero at some distance down the tooth, but before the pitch circle. This profile change (usually by grinding) is optimized for a particular transmission load. The tooth deflection is load-dependent, and we have, in the present work, three load levels: L0, L1, and L2.

Systematic wear is generated on each active flank, on either side of the pitch circle. This is because the sliding between the teeth in contact is small at the pitch line. The effect of this type of wear is the increase in the spectrum magnitude at the higher harmonics (at least the second and third ones) of the gear mesh frequency [1].

Localized pitting, like the one in the current work, and other local faults (spalls and cracks) generate a flat sideband spectrum with numerous sidebands of almost uniform and low level amplitudes. For comparison, we mention the impulsive excitation of the tested structure in experimental modal analysis (EMA) to measure frequency response function (FRF). In this scenario, a hammer with a stiff tip generates an impulsive force (with a short duration), resulting in a broad response in frequency [37]. This is particularly the case for impulsive-type signals that are typically the result of internal sharp impacts, for example, from local spalls in gears and bearings. Distributed faults will generate a modified time-domain vibration signal with a wider envelope associated with the fault zone in the time signal. This makes the corresponding envelope in the frequency domain narrower and higher in amplitude [1], with sidebands and harmonics that have high-level amplitudes close to the GMF. Some faults can also increase the amplitude of the GMF and its harmonics. One can conclude that various defects, their shapes, or the manufacturing errors of gears are encoded into the GMFs, harmonics, and their sidebands in the frequency domain.

Phase modulation is the deviation in phase (angular displacement) from the linearly increasing phase of the carrier signal. Frequency modulation is the derivative of phase modulation [1,9]. The frequency modulation of a carrier signal (with constant frequency) is the change in the frequency (angular velocity) of the carrier frequency according to the instantaneous amplitude of another function that encodes defects. The frequency modulation is not directly due to the changes in the rotation speed of the gears; the most significant part of it comes from the modulation in space of the tooth contact point, so that the frequency modulation would be present even if the gears were connected to infinite inertias so that their speed remained constant [1]. The amplitude of the sidebands is mathematically described by the Bessel function of the first kind [38]. In general, the sideband amplitudes decrease as the sideband pairs are farther away from the GMF’s harmonic, so one has to consider the significant sidebands.

Gear defects are imprinted in the measured vibration signal as a combination of the AM and FM modulation processes. For the mathematical formalization of the meshing (modulated) vibration, one can consider the ideal noise-free signal $x_{c}(t)$ to approximate the gear mesh frequency (of a gear pair with identical and equally spaced teeth) and its first *M* tooth-meshing harmonics caused by the time-varying (periodic) mesh stiffness *k*(*t*) function [36,39,40,41]. $x_{c}$ from Equation (2) represents the carrier frequencies in the modulation phenomenon:

(2) $$x_{c}(t)=\sum_{m=0}^{M}x_{m}\cos(2\pi z_{1}f_{p}mt+\phi_{m}),$$

where each harmonic component in the sum has constant amplitude and frequency, $z_{1}$ is the number of pinion teeth, $f_{p}$ is the rotation frequency of the shaft on which pinion P1 is mounted, $z_{1}f_{p}$ is the gear mesh frequency (an integer multiple of the rotation speed), $x_{m}$ is the amplitude of the *m-*th harmonic, $\phi_{m}$ the phase associated with the *m-*th harmonic, *t* is time, and the first term $x_{0}\cos(\phi_{0})$ is a constant. $x_{c}(t)$ is a component of a more general vibration signal x(t) of a measured gear mesh process of a pair of meshing gears.

For a defect (localized pitting, localized wear, crack, etc.) located on the pinion tooth flank, because of the localized stiffness change (in the gear pair), the vibration signal is modulated, in amplitude and phase, by the faulty tooth. The modulation frequencies are given by the shaft rotational speed ($f_{p}$) on which the faulty pinion P1 is mounted or its multiples, and the modulation functions are of the Fourier series form (Equations (3a) and (3b)) with *L* and *N* terms, respectively:

(3a) $$a_{m}(t)=\sum_{l=0}^{L}A_{m,l}\cos(2\pi f_{p}lt+\alpha_{m,l}),$$

(3b) $$b_{m}(t)=\sum_{n=0}^{N}B_{m,n}\sin(2\pi f_{p}nt+\beta_{m,n}),$$

where $A_{m,l}$, $B_{m,n}$ are the amplitudes of modulation functions at the corresponding sideband and $\alpha_{m,l}$, $\beta_{m,n}$ are, respectively, the phase at the *l-*th or *n-*th sideband of the amplitude-modulated signal around the *m-*th meshing harmonic. A simplification is considered, so the modulation is simultaneous for all the harmonics of the gear mesh frequency in the vibration signal. Hence, the initial phase $\alpha_{m,l}$ and $\beta_{m,n}$ is independent of the *m-*th harmonic, resulting in $\alpha_{m,l}=\alpha_{l}$ and $\beta_{m,n}=\beta_{n}$. The modulated vibration signal x(t) generated by a pair of meshing gears with a faulty tooth flank becomes of the form presented in Equation (4).

(4) $$x(t)=\sum_{m=0}^{M}x_{m}\left[1+a_{m}(t)\right]\cos\left[2\pi z_{1}f_{p}mt+\varphi_{m}+b_{m}(t)\right]=\sum_{m=0}^{M}\left[u_{1}(t)+u_{2}(t)\right],$$

where $u_{1}(t)$ is the modulated vibration signal caused by phase modulation and $u_{2}(t)$ is due to the amplitude modulation and its coupling with the phase modulation. $\varphi_{m}$, $\alpha_{l}$, and $\beta_{n}$ are random variables in the [0, 2π] interval. After algebraic manipulations [40] by using Jacobi–Anger expansion and the associated Bessel functions of the first kind, expressions that will be the sidebands’ source in the power spectrum, around the gear mesh frequency and its harmonics, are obtained.

The effects of AM and FM are inseparable in the resulting spectrum. The symmetrical families of sidebands (equally spaced around a center frequency) cumulate the effects of amplitude and frequency modulation. From the sidebands’ combination (AM and FM), the two amplitudes of a sideband pair can result asymmetrical about the fundamental frequency or their harmonics [1]. It is possible to have sideband amplitudes greater than the fundamental frequency, or even the case with an unnoticeable fundamental frequency from the spectrum [38]. In many cases, because of the low amplitude levels of the GMFs, their harmonics, and sidebands for incipient localized faults, a logarithmic scale of the spectrum is recommended.

The number and the amplitude of the sidebands are often correlated with the severity of the gear fault. As the deterioration progresses, the energy level between the sidebands and the center frequency tend to increase, and the apparition of the second pair of sidebands is observed. For localized defects on the gear from the modulation process, the resulting sidebands (at the faulty gear rotational frequency) with associated peaks are hardly discernible in the spectrum. Cepstrum analysis presents advantages, namely, through quefrencies that collect peaks, and thus the defect is more easily observable.

Condition indicators that consider regular mesh components and sidebands have been developed in order to evaluate the gear faults. One can mention the sum of the amplitudes of the GMF and its total harmonics in the vibration signal spectrum, Sideband Power Factor, Amplitude of Sidebands, dimensionless Amplitude of Sidebands, and others. In [42], several standard diagnostic parameters were modified (some of them in relation to the TSA approach) in order to account for a crack in the epicyclical (planetary) gearbox of a main transmission with a five-planet epicyclical gear train of an Army UH-60A Blackhawk Helicopter.

On the TSA approach, the so called “regular” mesh components (which refer to the fundamental shaft frequency, the fundamental gear meshing frequency, its harmonics, and their first-order sidebands) are excluded from the averaged vibration time signal. The first-order sidebands, in some cases, are due to the run-out of the gear because of machining or assembly inaccuracies, and thus can be considered regular components [42].

The gear tooth surface quality could impair the capability of detection of local faults, like, for example, in [10], where angular resampling and synchronous average (SA), followed by a spectral analysis of the SA signals, are used to extract fault indicators and frequency parameters.

In order to extract the modulating signal, which is of interest in gear fault detection, the demodulation of the real measured signal (where the carrier signal is mixed with the modulating signal) is applied. Widely used demodulation methods for shafts with constant rotational frequency are FFT-based demodulation methods, Hilbert transform demodulation analysis, and energy operation demodulation analysis [1,43].

In [44], a data-driven method to automatically generate system health indicators is proposed. Spectral peaks are detected from successively acquired signals and grouped in structure as harmonic series or modulation sidebands. A time–frequency tracking operation is applied to the available signals: the spectral peaks and the spectral structures are tracked over time and grouped in trajectories, which will be used to generate the system health indicators. The method is validated on a wind turbine test rig.

Gear statuses with defects (a tooth crack and a broken tooth) versus gears without faults are separated in [45], where the concept of time–frequency entropy based on the Hilbert–Huang transform (HHT) is defined. The degree of disorder of energy distribution in the vibration signal with non-stationary characteristics given by gear faults is observed. The idea of extra energy occurring in the sidebands in addition to energy from the gear meshing frequency and harmonics when gear defects occur is emphasized.

The sidebands are of paramount importance in classical gear fault detection, and as will be shown in what follows, features from families of GMFs and their sidebands employed in machine learning provide good results with less effort and specialized knowledge.

In the proposed defect detection method, most of the features are extracted using MATLAB R2023a built-in functions, followed by multi-class classification with the machine learning MATLAB toolbox. One approach for fault detection is based on the parameters from time-domain and frequency-domain statistics. The second one, of primary importance, relies on features related to the GMF, its upper harmonics, and the associated sidebands.

## 3. Test Rig, Data Acquisition, and Data Processing

In this section, the test rig and the vibration data acquisition system employed in the experiments are presented. The preprocessing of the acquired signals, a stage prior to feature extraction, and the defects of the input shaft pinion of the first stage of the speed reducer are described.

### 3.1. Test Rig

**Figure 1:**
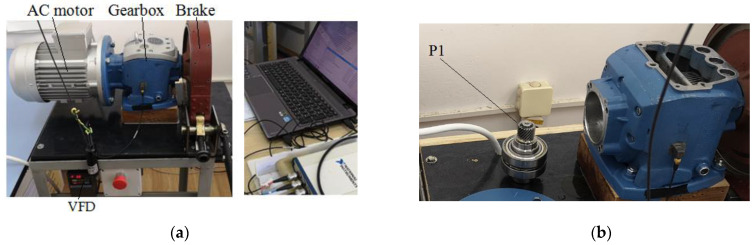

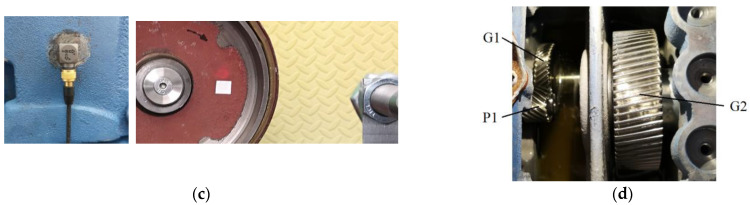
(**a**) Overview of the test rig and acquisition system; (**b**) input shaft with pinion P1 and two bearings; reducer top cover is removed; (**c**) triaxial accelerometer and tachometer (pointing to the friction disc); (**d**) top view of reducer with top cover removed.

The experimental test rig comprises a three-phase AC motor, a variable frequency drive (VFD) as a motor speed regulator, a speed reducer gearbox, and a dry friction brake, along with the acquisition system (Figure 1a).

**Figure 2:**
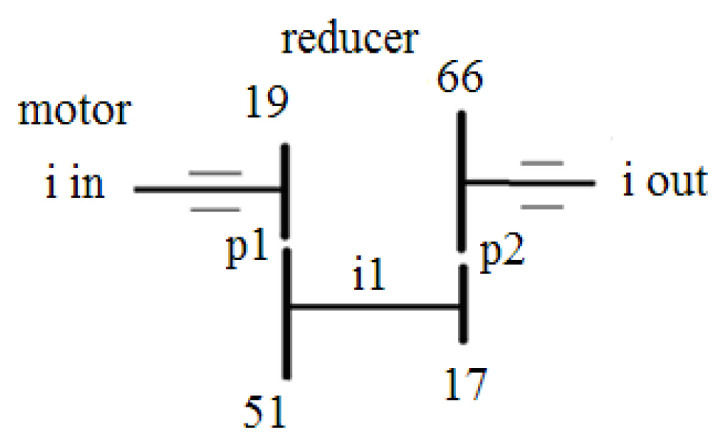
Speed reducer kinematic chain.

The pinion P1 under observation, mounted on the input shaft, can be seen in Figure 1b, where the reducer top cover is removed. On the lateral surface of the speed reducer cast iron casing, a triaxial B&K piezoelectric accelerometer with integrated electronics has been bonded (Figure 1c). The accelerometer’s X axis points downward and is perpendicular to the reducer’s base. The Y axis is parallel to the reducer input shaft and points to the right, whereas the Z axis is perpendicular to the lateral reducer’s bonding surface. Figure 2 depicts the schematization of the two stages of the speed reducer with the fixed parallel shafts and two gear pairs, p1 (P1,G1) and p2 (P2,G2).

The actual speed reducer ratio *ir* = *i_in_*/*i_out_* is 10.421, and the relation between the input and output turning speeds (derived from the teeth numbers of the two gear pairs) is expressed in Equation (5).

(5) $$i_{in}\times \frac{19}{51}\times \frac{17}{66}=i_{out}$$

The National Instruments USB-4431 (four input channels simultaneously sampled, 24-bit resolution and one output channel) dynamic signal acquisition board and a LabVIEW application are used for the acquisition. The first three analog input channels are connected to a triaxial accelerometer, and the fourth input channel records the tachometer pulses.

In the present study, the AC motor drives the speed reducer input at three nominal rotation speeds of 1350 rpm, 1375 rpm, and 1400 rpm. For each speed, three different load levels, the unloaded state (L0) and two different friction levels (L1 and L2), are imposed by using the friction brake with fixed lever positions. These nine combinations are imposed on each of the four condition states under observation. The defect detection task is modeled as a multi-class classification problem, with the four classes (D1, D2, D3, and D4) corresponding to the gearbox states, regardless of the speed and the load. D1 (low oil level in reducer) is characterized by poor lubrication, and the wheel G1, which is positioned under the pinion P1, is immersed in lubricant only with the teeth underneath. D2 is the normal state without defect. State D3 corresponds to a localized pitting on a pinion tooth flank. The D4 state presents a localized wear on a pinion tooth flank. The D3 and D4 states are described in what follows, and in more detail in [31].

### 3.2. Localized Pitting and Localized Wear Defects on the Helical Pinion

A helical gear is able to carry a larger load than a spur gear and operates more smoothly and more quietly because more tooth pairs are in contact during meshing. The contact on the active flank pair is realized along the contact line and the sum of the length of the contact segments (which varies slightly during the gear meshing cycle) is proportional to the capable gear pair thrust load. A localized pitting defect (which is our case) on a helical gear has a diminished effect in the measured vibration signal. During meshing, the sliding velocity between the active flanks (the relative tangential velocity of the tooth profiles) is important compared with the relative velocity (which is theoretically zero) at the pitch diameter where the pitting defect observed in the experiment is approximately located. Two flank surface faults, namely, localized pitting and localized wear belonging to pinion P1 (of p1 gear pair), are observed in the experiments.

Gear tooth pitting, which can occur in macro or micro forms, is the most prevalent type of failure. Macro-pitting refers to pits larger than 1 mm in diameter. Usually, pitting develops in a small area at or just below the pitch line.

**Figure 3:**
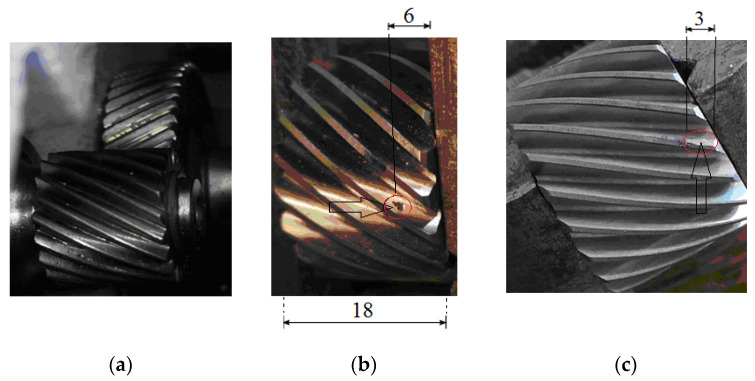
(**a**) p1 (P1,G1) gear pair; (**b**) pitting defect on pinion P1; (**c**) localized wear on P1.

Pitting is caused by the contact fatigue of the steel gears’ tooth flanks, which removes material from the tooth surface. The gear material fatigue life is given by the S-N curve (Woehler curve). Lubrication (oil viscosity and temperature), working loads, specific sliding between contact flanks, and flank surface roughness and shape have an impact on the process. A high stress gradient over a very short distance of the unpitted surface, as a result of the hydraulic pressure of the oil acting in the microcracks (later in cracks), during the mesh causes the cracks to expand into pits. Pitting starts near the pitch point, where a high frictional force is present (due to the low sliding speed), and it is able to spread to neighboring regions, usually taking the form of a shell. In the experiments, the localized pitting defect belongs to the active flank of one tooth of the P1 pinion (with 19 teeth), which is meshing with wheel G1 (with 51 teeth). In Figure 3, the defect can be observed as placed at the mid-width of G1 and located approximately 6 mm away from the P1 pinion’s right side. The shape of the defect is approximately triangular, with an inscription in a circle of 1 mm, and the maximum depth is about 0.2 mm.

When a tooth pair of the helical gear begins to engage, the contact starts gradually from one end of the teeth and maintains the contact as the gear rotates in full involvement. The material loss from the engaging tooth flanks due to sliding and rolling motions is named wear on flanks. In the current experiment, the wear defect is 3 mm long along the helical tooth belonging to pinion P1, with a maximum width of 0.9 mm (Figure 3). This defect involves a transverse change of the profile slope (chamfering) at the addendum of the unhealthy tooth, with the effect of shortening the contact line when the area of the defective pinion tooth is engaged with the G1 wheel. The helical gear transmission ratio for the localized wear fault (as for the localized pitting) is less sensitive in comparison to spur gear. This is due to the increased length of contact lines associated with the number of tooth pairs found in contact (contact ratios). In terms of GMF harmonics, it has been noted that the higher order harmonics are more sensitive to tooth wear in comparison to the lower ones.

### 3.3. Data Acquisition and Preprocessing

A LabVIEW application was written to acquire the three-axis accelerometer signals as well as the corresponding tachometer signals. Three rotation speeds were chosen for each load level from the three different loads, yielding nine files (acquisition records) per health state and a total of thirty-six files for four health states. MATLAB R2023a was used to retrieve analyzing records from these files and process them either individually or as groups. Each analysis record contains three lines, one for each accelerometer axis (X, Y, and Z).

The tachometer measures the rotational speed of the reducer output, *i_out_*, and then uses the speed reducer ratio in Equation (1) to calculate the pinion P1 rotational speed, *i_in_*. The three reducer output rotational speeds used were 2.16 rps, 2.20 rps, and 2.24 rps, which were close to the motor’s maximum capabilities. Table 1 shows the pinion and its corresponding AC motor speeds.

The sampling frequency of the signal acquisition is F_s_ = 10 kHz and the duration of one acquisition record is 240 s. The acquisition records with load L2, speeds v2, and v3 for the classes D2, D3, and D4 have a shorter duration of 120 s. In an analyzing record (observation), we propose an integer number of pinion P1 rotations, namely, 18 rotations, which means about 1.8 rotations at the reducer output shaft, regardless of motor velocity. Considering the constant acquisition sampling frequency (F_s_), for a lower input shaft rotation speed (longer time per revolution), a larger number of samples are acquired for a pinion (P1) complete rotation, as follows: 444.26 samples/rotation for v1 rotation speed [rps], 436.18 samples/rotation for v2 rotation speed [rps], and 428.39 samples/rotation for v3 rotation speed [rps].

An analyzing record (observation) is saved in a Comma Separated Values (csv) file. Each csv file contains three rows, one row for each accelerometer axis. The number of samples in an acquisition record is divided by the number of samples in 18 rotations. A total of 36 folders (with csv files), associated with 4 states × 3 loads × 3 velocities, are generated. The files pertaining to the same state are collected together. The dataset used further in feature extraction contains a total of 9902 observations.

Features are extracted from each observation (a csv file), yielding 2591 instances for class D1 and 2437 instances for each of the other three classes, D2, D3, and D4. Two approaches to fault classification are proposed in what follows. In the first approach, statistical features in time and frequency domains are used to represent the vibration signals, and then machine learning techniques are applied. In the second approach, the band spectrum powers and the peak amplitudes for GMF harmonics and sidebands are calculated and used as features in classification.

## 4. Methodology

**Figure 4:**
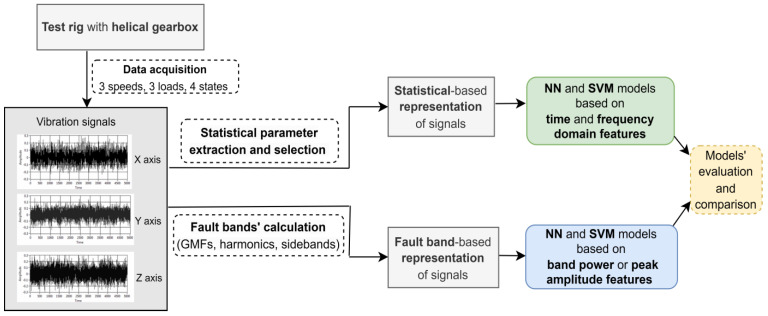
Methodology.

This section describes the proposed methodology (Figure 4) applied in the present study. The data acquisition process was presented in Section 3.3. Two different types of representations for the vibration signals are used as input to the SVM (Support Vector Machine) and NN (Neural Network) classification models [46]. These models, briefly described in Section 4.1, are two of the most popular and efficient machine learning techniques employed in multi-class classification and have proven to be the best performing models in the experiments conducted in the current study. The first representation contains statistical parameters extracted from the vibration signals (Section 4.2). The second one, introduced in Section 4.3, is based on the power spectrum bands for GMF harmonics and associated sidebands.

### 4.1. SVM and NN Classification Models

The standard SVM algorithm was introduced to solve the binary classification problem by using a maximum-margin hyperplane that linearly separates the data points from the two classes. If the boundaries of the classes are non-linear, kernel functions are applied to transform the features from the initial space into a higher dimensional feature space where the two classes might be linearly separable. The most commonly used kernels are polynomial (quadratic and cubic) and Gaussian. The multi-class (*n* classes) classification problem is reduced to more binary classification problems using one of the two approaches: *one-versus-one* and *one-versus-all*. The *one-versus-one* approach uses a binary classifier for each pair of classes, so a total of *n*(*n* − 1)/2 binary SVMs. In the *one-versus-all* approach, *n* binary SVMs are utilized to solve *n* binary classifications. In such a binary classification, the task is to discriminate between an initial class (from the *n* classes) and a large class containing the remaining *n* − 1 initial classes. The advantages of the SVM classifier include efficiency in handling high-dimensional data and small datasets, robustness to noise, and good generalization performance.

A Neural Network is a structure of interconnected neurons grouped in layers, inspired by the human brain, with the goal of learning from input data to recognize patterns and classify the data. The information traverses the layered structure from the input layer to one or more hidden layers and to the output layer. A neuron calculates a weighted sum of the information provided by all the neurons in the previous layer and then applies an activation function to pass information to the next layer. The weights associated with the connections between neurons are updated during the iterative training process based on the comparison between the predicted and the desired output in each iteration by applying backpropagation. Using non-linear activation functions (ReLU-rectified linear units, sigmoid, and tanh), the classification problem of non-linearly separable data is solved. The hidden layers work as feature engineering steps by capturing hidden properties of the input data at different levels of abstraction.

The architecture of a shallow, single-hidden-layer NN used for *n*-class classification consists of the following layers: (1) an input layer with *m* neurons, corresponding to the input representation; (2) a hidden layer with *h* neurons, a fully connected layer that uses a non-linear activation function; (3) an output layer, fully connected, with *n* neurons; (4) a softmax layer with *n* neurons containing the membership probabilities (calculated with *softmax*, the normalized exponential function) for the *n* classes; and (5) a classification layer, corresponding to one-hot encoding of the predicted class. The hidden layer corresponds to an abstract representation of the input data, a representation used further in classification. Due to their simple architecture, shallow NNs converge faster during training, are less prone to overfitting, and provide good generalization for small datasets.

Both SVM and shallow NN classifiers can solve the classification problem of non-linearly separable data and achieve comparable accuracy on the same dataset. From a training perspective, SVMs are faster to train than NNs.

### 4.2. Statistical Parameters in Time Domain and Frequency Domain—Extraction and Selection

In the first approach, 23 statistical parameters (Table 2), 16 in the time domain and 7 in the frequency domain, for each of the three accelerometer axes, are proposed to be used as features in the defect detection task. We aim to answer research question RQ1 and identify the most relevant statistical parameters that best discriminate between the four condition states under observation.

The initial set of statistical features, denoted as Fs_69, contains 23 parameters calculated from the vibration signal for each axis, totaling 69 features for all three accelerometer axes. This set of 69 features extracted from each observation of the initial dataset is then analyzed with the MATLAB *relieff* function [47] to select the most important features. Based on the selected features, the statistical representations of the signals are generated and further used to build NN and SVM models. These models are employed to solve the defect detection task as a multi-class classification problem.

### 4.3. Fault Bands’ Calculation—GMF, Harmonics, and Sidebands

To answer research question RQ3, the signal representation is based on the GMF harmonics and associated sidebands.

**Figure 5:**
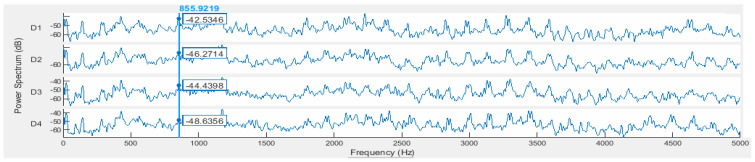
A sample logarithmic scale power spectrum for each class, on Y axis and v1 velocity.

In the power spectrum associated with each analyzing record (csv file), for the first gear pair (p1), the fundamental pinion shaft frequency ($f_{p}$), the fundamental gear mesh frequency (GMF), and its harmonics can be considered regular gear pair components. One can add to this group the first-order pair of modulation sidebands, which are generally due to the run-out of the gear because of machining or assembly inaccuracies. A sample logarithmic scale power spectrum for each class (D1, D2, D3, and D4), on the Y axis and for v1 rotation speed is shown in Figure 5. The cursor is positioned at the second harmonic of the GMF (855.38 Hz) of the p1 gear pair. The frequency resolution (df) is 1.221 Hz.

**Figure 6:**
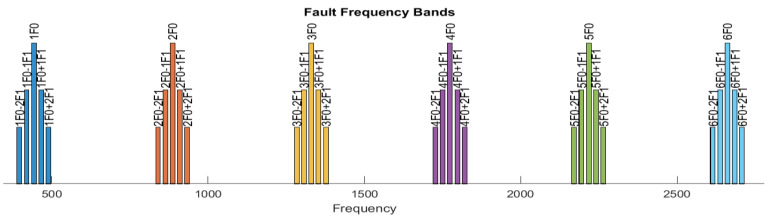
Six GMF harmonics and associated sidebands, along the frequency axis ([Hz]).

The MATLAB R2023a *faultBands* function served to derive the frequency bands associated with the GMFs and their sidebands. The input parameters of the function are the fundamental value of the GMF, the number of GMF harmonics, the rotational speed of the pinion P1 shaft, the number of sidebands for each GMF harmonic, and a proposed value for the band width. The fault frequency band width (15 Hz) was selected to cover a larger interval, considering localized defects. All frequency bands are depicted in Figure 6. The frequency is expressed in Hz. For example, 2F0 is the notation of the band with a center frequency equal to 2 × F0, where F0 is $f_{p}$ × $z_{1}$ ($f_{p}$ can be v1, v2, or v3), while 2F0+2F1 is the notation of a sideband with a center frequency equal to 2 × F0 + 2 × F1, where F1 is $f_{p}$.

Peak amplitude, peak frequency, and band power for each frequency range specified in the thirty (30) fault frequency bands of interest (returned by the *faultBands* function) are calculated using the MATLAB function *faultBandMetrics*. The band power in angular frequency band [*ω*1, *ω*2], using the power spectral density (non-negative) function $S_{a}(\omega )$, is calculated with Equation (6):

(6) $$P_{band,a}=\frac{1}{\pi }\int_{\omega_{2}}^{\omega_{1}}S_{a}(\omega )d\omega ,$$

where *ω* is the circular frequency [rad/s] and the index *a* is the X, Y, or Z axis.

**Figure 7:**
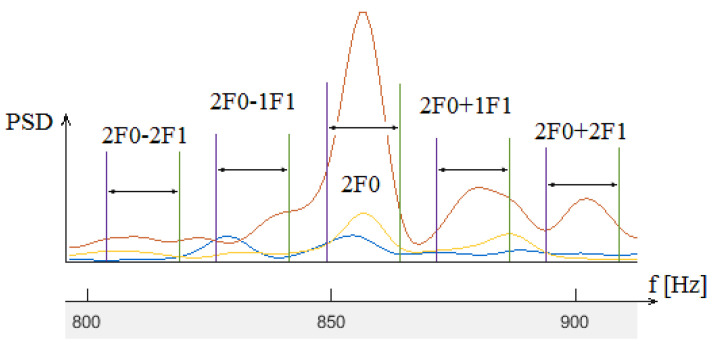
Power spectral density for the X (yellow), Y (brown) and Z (blue), axes, second GMF harmonic (2F0), and two sideband pairs.

A sample graph representing the power spectral density for the X, Y, and Z axes, the second GMF harmonic (855.38 Hz) at the lower (v1) speed, and two sideband pairs is depicted in Figure 7. Five frequency bands can be observed. The graph shows a prominent peak for the second GMF harmonic, two soft peaks for the two right sidebands (2F0+1F1, 2F0+2F1), and possibly low and hidden peaks for the two left sidebands (peaks of power spectrum increased on the right side and partially canceled on the other side).

In the present paper, six GMF harmonics are considered, with both the peak amplitude in the fault band and the fault band power. In order to also evaluate the relevance of the GMF sideband pairs in the defect detection task, six feature sets based on fault bands are proposed, and they will be described in what follows. The notations from Figure 6 are used in the features’ names.

We take from the power spectrum a set of six fault *bands* for the first six GMF harmonics, which are 1F0, 2F0, 3F0, 4F0, 5F0, and 6F0; hence, the bands are identified by the frequency in the middle of the band.

- BP_GMF is the set of 18 features representing the *band powers* for the previous six fault bands and for all three accelerometer axes.
- PA_GMF consists of 18 *peak amplitudes* for the same six GMF fault bands and for all three accelerometer axes.

For each GMF harmonic, three fault bands are considered, which are the central GMF harmonic and the first pair of sidebands, i.e., a total of eighteen fault bands for six harmonics:

(1F0-1F1, 1F0, 1F0+1F1), (2F0-1F1, 2F0, 2F0+1F1), …, (6F0-1F1, 6F0, 6F0+1F1)

- BP_GMF_1sb contains 54 features representing the *band powers* for the previous 18 fault bands and for all three accelerometer axes.
- PA_GMF_1sb is the set of 54 features representing the *peak amplitudes* (instead of *band powers*) for the same fault bands as in BP_GMF_1sb.

For each GMF harmonic, five fault bands are considered, which are the central GMF harmonic and the first two sideband pairs, resulting in thirty fault bands for six harmonics:

(1F0-2F1, 1F0-1F1, 1F0, 1F0+1F1, 1F0+2F1), …,

(6F0-2F1, 6F0-1F1, 6F0, 6F0+1F1, 6F0+2F1)

- BP_GMF_2sb contains 90 features representing the *band powers* for the previous 30 fault bands and for all three accelerometer axes.
- PA_GMF_2sb is the set of 90 features representing the *peak amplitudes* (instead of the *band powers*) for the same 30 fault bands as in BP_GMF_2sb.

All these feature sets are used further to build NN-based and SVM-based classification models that are employed to perform the four-class classification and to solve the proposed defect detection task.

## 5. Experiments and Discussion

This section presents the experiments performed in MATLAB R2023a on the initial dataset with 9902 observations. Of these, 2591 observations are for class D1 (low oil) and 2437 observations for each of the other three classes: D2 (normal state), D3 (localized pitting), and D4 (localized wear). Two types of signal representations and two classification models, SVM and NN, are comparatively used to answer the research questions proposed in the Introduction.

For the SVM model, we tried different configurations, combining the following hyperparameters: (a) standardized data; (b) kernel function: Linear/Quadratic/Cubic/Gaussian; (c) multi-class coding: one-versus-one/one-versus-all; and (d) box constraint level: 1/2.

The Neural Network (NN) model was applied to standardized data, with ReLU activation function (ReLU(x) = max{0,x}), Softmax function for prediction, and two different configurations for the single hidden layer: 25 neurons (NN-medium) and 100 neurons (NN-wide).

The best suitable models for our task were found to be SVM-cubic (one-versus-one, box constraint level: 1) and NN-wide, in all experiments performed.

All the classification models are built on 85% of the dataset and tested on 15%, according to Table 3, and using several feature sets.

The overall performance of the proposed models is assessed using the testing Accuracy metric, which is computed as the percentage of examples correctly identified out of all the tested instances. At the class level, the evaluation metrics used are Precision, Recall, and their harmonic mean, F1-score [48].

### 5.1. Defect Detection Based on Statistical Features

The parameters proposed in Section 4.2 are used for the statistical representation of the vibration signal. An important step prior to classification is the selection of the most informative features that best distinguish between the four defects (states).

The applied feature selection process is based on ranking the features in a supervised manner, according to their importance as predictors in the four-class classification. The *relieff* function [47] from MATLAB provides the features’ weights in the interval [−1, 1] and the corresponding ranking. A positive weight value close to 1 expresses the high relevance of that feature in classification. The Pearson coefficient [49] is used to identify linear relationships between pairs of features, so it helps to select only independent features.

For the initial dataset consisting of 9902 observations, a matrix, denoted by *mat69* (9902 lines and 69 columns), is generated. Each line contains 23 statistical parameters (Table 2) for each of the three axes, extracted from that observation, totaling 69 features.

The symmetric matrix with the Pearson coefficients between all pairs of the 69 features is calculated from *mat69*. As was expected, the pairs in the set of features {*mean*_*abs*, *std*_*dev*, *rms*, *mG*}, for each axis, are very highly correlated, with a coefficient > 0.99. Therefore, just one feature, *mean*_*abs*, will be kept, as it is the most relevant one based on the features’ ranking provided by *relieff*.

The *relieff* function is applied to the matrix *mat69* and the column of classes corresponding to the output defects (states). Based on the features’ weights, from the initial set of 69 features, 22 of them were selected in the set Fs69_sel22, to be used further in data visualization and then as predictors in classification. This set contains six parameters extracted from each of the three axes and two parameters (peak2peak and mom3) calculated from the signals measured on two axes. The order of the parameters in Fs69_sel22 corresponds to the ranking provided by the *relieff* function.

Fs69_sel22 = {zcrate_Y, Mfreq_Y, apparent_Y, mean_abs_Y, lyapExp_Y,
    mean_abs_Z, Mfreq_Z, Mfreq_X, zcrate_X, lyapExp_X, zcrate_Z,
      mom4_Y, lyapExp_Z, apprEnt_X, mom4_Z, mean_abs_X, apprEnt_Z,
   peak2peak_Z, mom4_X, mom3_X, peak2peak_Y, mom3_Y}.

A detailed analysis of the features’ scores (weights) shows that the statistical parameters extracted from the signal measured on the Y axis are more relevant than the corresponding parameters extracted from the other two axes. This observation is consistent with the result obtained in [31], where the features learned by a 2D-CNN from the signal measured on the Y axis proved to better discriminate between the same four defects (classes) than the features extracted from the signal measured on the X or Z accelerometer axes.

**Figure 8:**
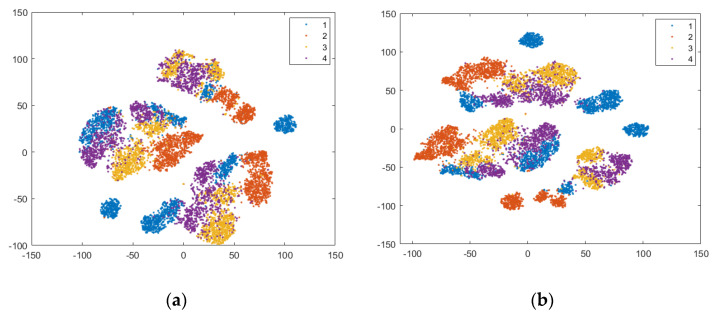
Two-dimensional t-SNE projection of the initial dataset in the feature space: (**a**) Fs69_sel22, and (**b**) Fs72_sel25.

To illustrate how separable the four classes (defects) are, a 2D graphical representation of the initial dataset in the feature space defined by Fs69_sel22 is helpful. The dimensionality is reduced using an unsupervised method called t-SNE (t-Distributed Stochastic Neighbor Embedding) [50], which maintains the clusters from the original high-dimensional space in the low-dimensional space. In Figure 8a, the 2D t-SNE projection of the initial dataset in the feature space specified by Fs69_sel22 is depicted. The best detectable classes are D2 (red) and then D1 (blue). We notice that classes D3 (yellow) and D4 (magenta) are mixed together at the boundaries of their clusters, as well as with D1.

Based on this visualization, very good classification results are not expected, and a new feature, paGMF (peak amplitude of GMF), is proposed to improve defect detection. This non-statistical feature is important, because it represents the spectrum amplitude at the meshing frequency of the reducer first gear pair. The values of paGMF calculated on all three axes are added to Fs69 to obtain Fs72, and they are also added to the 22 selected statistical features to produce the set Fs72_sel25.

According to the scores provided by the *relieff* function, these new features are relevant in prediction. In Figure 8b, we observe a small improvement in class separability for all classes, with better delimited clusters for D1 and D2.

To answer research questions RQ1 and RQ2, experiments were conducted on four sets of features, namely, Fs69, Fs69_sel22, Fs72, and Fs72_sel25 (Table 4), comparatively using SVM and NN as classification models. Ten runs were performed on each experiment, defined as a combination of a feature set and a classifier. In a run, the initial dataset was randomly split into 85% for *training* (five-fold cross-validation) and 15% for *testing*, in order to provide a fair comparison of the classification results. The proposed feature sets were extracted from the training and testing sets (refer to Table 3), which were then used in the corresponding experiments.

The performance of an experiment is provided as a confidence interval, $CI=ma\pm 1.96\sigma /\sqrt{n}$ [51], at the 95% confidence level of the mean accuracy (*ma*) over *n* = 10 runs, with σ being the standard deviation. Table 5 reports the classification results for all eight experiments.

The feature selection step proved to be beneficial in improving the classification. The accuracy increases from Fs69 to Fs69_sel22 and from Fs72 to Fs72_sel25 in both classification models. We also note the positive impact of the new feature (paGMF) in the defect detection task, expressed by the very good accuracy (>98%) achieved by both classifiers, slightly more than 2% higher than the accuracies obtained using only the 22 selected statistical features.

To illustrate the practical importance of the paGMF feature in tooth flank quality detection, we present in Table 6 the confusion matrices and calculated metrics (Precision, Recall, and F1-score) in a run of the NN-wide classifier, using (a) Fs69_22sel and (b) Fs72_25sel. The number of tested instances correctly predicted is highlighted in blue on the diagonal, while the instances wrongly classified are marked in beige. In the feature space defined by Fs72_25sel, there is a 2.25% improvement in overall accuracy, and the F1-score increases by 3.28% for D4, 2.88% for D1, and 2.8% for D3. All these results are consistent with the 2D t-SNE projections in Figure 8.

**Figure 9:**
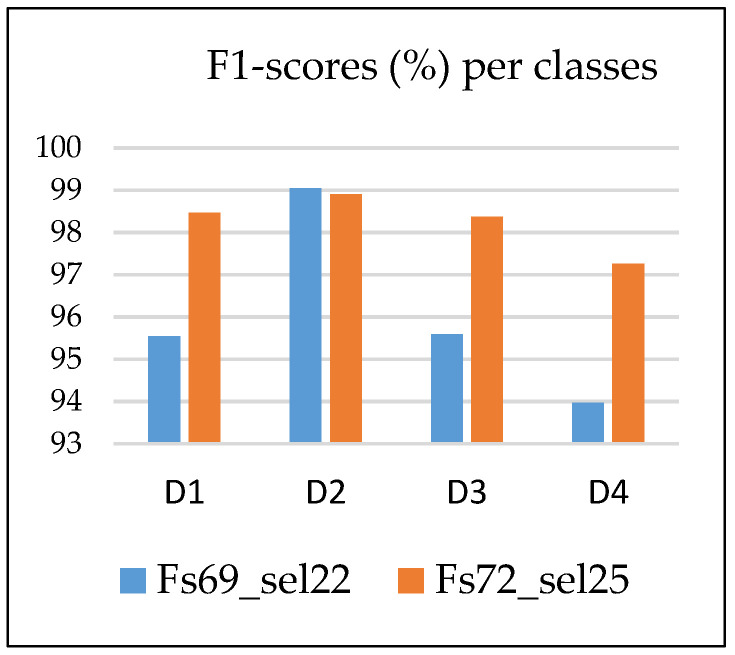
F1-scores per class in Fs69_sel22 versus Fs72_sel25 feature spaces.

The chart in Figure 9 presents the F1-scores per class in the Fs69_22sel and Fs72_25sel feature sets, indicating a significant improvement in the detection of classes D1, D3, and D4 when using the paGMF feature (calculated on each of the three axes) in classification. Class D2 (normal state) remains the best detectable class, and D4 is the least detectable.

### 5.2. Defect Detection (Classification) Based on GMF, Harmonics, and Sideband-Related Features

Based on the idea that the defects on gear active tooth flanks are captured in the amplitude and phase modulation of the vibration signal, six harmonics of the GMF and two sideband pairs are used to extract specific features for multi-class classification.

To answer research question RQ3, experiments were carried out on the six sets of features described in detail in Section 4.3 and presented in Table 7. BP_GMF, BP_GMF_1sb, and BP_GMF_2sb contain the band powers for harmonics, harmonics with the first pair of sidebands for each, and harmonics with two pairs of sidebands, respectively. PA_GMF, PA_GMF_1sb, and PA_GMF_2sb contain the peak spectrum amplitudes for the same combinations of harmonics and sidebands as the three sets previously described.

The efficiency of the proposed feature sets in defect detection was compared using SVM and NN classification models. The performance results obtained in the experiments are presented in Table 8 as confidence intervals at the 95% confidence level of the mean accuracy over 10 runs. In a run, the initial dataset was randomly split into 85% for training (5-fold cross validation) and 15% for testing (refer to Table 3), in order to provide a fair comparison of the classification results.

**Figure 10:**
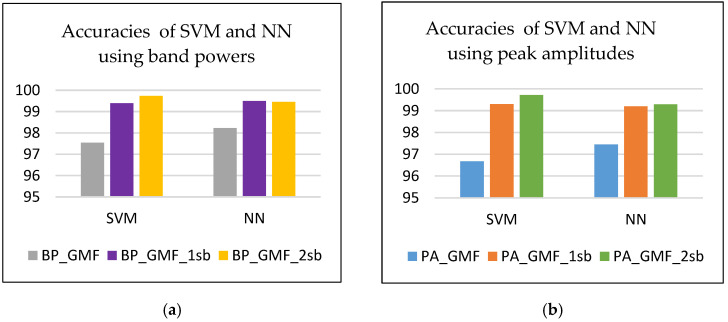
Accuracies achieved by SVM and NN using (**a**) band powers and (**b**) peak amplitudes.

The accuracy values in Table 8 reveal that the features based on GMF and sidebands are suitable for detecting the four defects (states) under observation. The smallest feature sets, those with only 18 features, representing band powers (BP_GMF) or peak spectral amplitudes (PA_GMF) of the six harmonics on all three axes, proved to be relevant in distinguishing the classes. As features, the band powers are slightly more informative than the peak amplitudes. Information (band powers or peak amplitudes) from the first pair of sidebands increases the accuracy (by a minimum of 1.26% and a maximum of 2.62%) of both classification models (Figure 9). A slight improvement is observed in the SVM model by adding powers/amplitudes from the second pair of sidebands (Figure 10). In the NN model, the features provided by the second pair of sidebands, such as band powers or peak amplitudes, are not relevant in the present defect detection.

Experiments with the reunion of BP_GMF_2sb and PA_GMF_2sb sets, which contain the band powers and the peak amplitudes, totaling 180 features, did not result in significant improvements in classification. The SVM model achieved an accuracy of 99.85%, while the NN model achieved 99.53%.

A comparative classification analysis at the class level, in the feature sets containing the band powers (BP_GMF, BP_GMF_1sb, and BP_GMF_2sb), was further conducted. In Table 9, Table 10 and Table 11, we notice the consistency of the classification results (one run of SVM-cubic) with the 2D t-SNE projection of the initial dataset in the corresponding feature space. For a fair comparison, the same split for training (85%) and testing (15%) of the initial dataset was used in the runs of the SVM model.

In all three t-SNE projections, there is a separation of instances according to load (L0, L1, and L2), and in the corresponding regions, there are clusters, relatively delimited, for the classes (states). Separability between classes is increased by adding features, especially those from the first pair of sidebands for each harmonic, which also help to distinguish between different speeds (there are three speeds for a load). For L0, in all three feature spaces, there is an overlap of clusters corresponding to classes D3 (yellow) and D4 (magenta), which is consistent with the confusion matrices and the classification results, with these classes having the lowest F1-scores. A possible reason could be the fact that with the tooth flank contact for L0 being weak, the two defects (D3 and D4) are not well imprinted in the vibration signal. In the BP_GMF_2sb feature space, there is a clear separation of all four classes for L1 and L2, but for L0, only D1 (blue) and D2 (red) are very well delimited.

**Figure 11:**
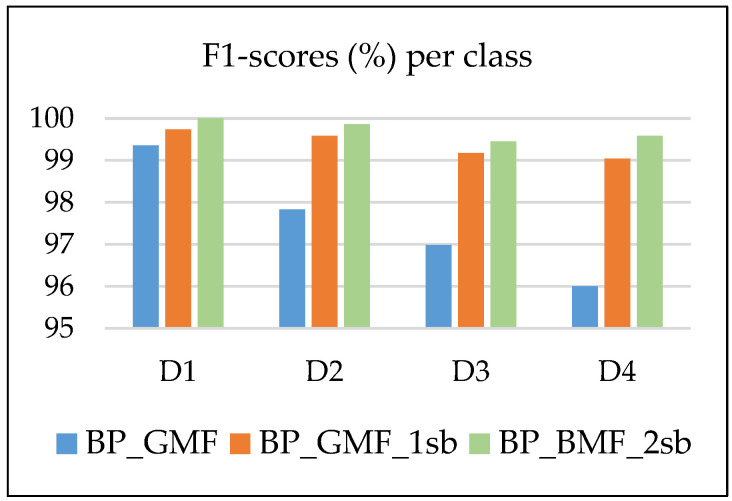
F1-scores per class in BP_GMF, BP_GMF_1sb and BP_GMF_2sb feature sets.

Figure 11 comparatively shows the F1-scores per class from Table 9, Table 10 and Table 11. We notice important classification improvements for the classes D4 (3%), D3 (2.15%), and D2 (1.76%) by adding as features the band powers corresponding to the first pair of sidebands for each harmonic. By using the band powers of the second pair of sidebands for each harmonic as additional features, the F1-scores increase by at most 0.5% (with the greatest value observed for D4). In all three feature spaces, the best detectable class is D1, followed by D2, and then D3 and D4.

The best performing classification model obtained in experiments can be used in the deployment stage. A final model is built by training a SVM (cubic kernel and one-versus-one approach for four-class classification) on the full set of 9902 observations and using the BP_GMF_2sb feature set. This set comprises, for each axis, the band powers of six harmonics and two pairs of sidebands associated with each of them, for a total of 90 features on all three axes. This model is exported and will be applied for future defect predictions.

Table 12 comparatively presents the defect classification models’ performances applied to the same dataset of vibration signals recorded on all three accelerometer axes and using various feature sets. The conclusion is that the band powers of six regular GMF harmonics and the first two pairs of associated sidebands for each harmonic are the most relevant features in detecting the four condition states of the helical gears.

## 6. Conclusions and Future Work

A test rig with a speed reducer gearbox with helical gears was employed for gear fault detection. Four condition states were under observation: D1—low oil level in the speed reducer; D2—normal state; D3—localized pitting on a pinion tooth flank; and D4—localized wear on a pinion tooth flank.

Raw vibration signals, measured using a triaxial accelerometer for three different working speeds and three load levels for each speed, were used to detect the condition states of the gearbox. Statistical-based representations and fault band-based representations of vibration signals were proposed as input to the SVM and NN classification models.

The first research question, RQ1, was answered by selecting the 22 most relevant time-domain and frequency-domain statistical features that discriminate between the four states, regardless of load level or speed level on the test rig. An NN-wide model achieved the highest accuracy of 96%. Experiments using the previously selected statistical features and spectrum peak amplitudes at the meshing frequency of the speed reducer first gear pair on all three accelerometer axes showed a 2% improvement in accuracy, which is the answer to the second research question, RQ2.

To address research question RQ3, several sets of features were used. These features represent the band powers or/and peak amplitudes (from the vibration spectrum) on frequency bands associated with GMFs, harmonics, and sidebands. The best performing model, with a testing accuracy of 99.73%, was built on the SVM model and utilized the band powers for six GMF harmonics and the first two sideband pairs on all three axes as features.

Future work will observe a larger palette of gear defects in both stages of the speed reducer with helical gears for various loads and velocity combinations. Another direction of further research is the use of LSTM in defect prediction, a deep learning model that learns long-term dependencies between timesteps of data.

## Figures and Tables

**Table 1:** Pinion P1 and AC motor rotation speeds.

Nominal Speed Values	v1	v2	v3
Reducer taho velocity: *i_out_* [rpm]	129.6	132	134.4
Reducer velocity: *i_out_* [rps]	2.16	2.20	2.24
P1 (AC motor) speed: *i_in_* [rps]	22.51	22.93	23.34
P1 (AC motor) speed: *i_in_* [rpm]	1350.4	1375.4	1400.4

**Table 2:** Statistical parameters.

Time-Domain	Time-Domain	Frequency-Domain
Mean of absolute value (mean_abs)	4th momentum (mom4)	Spectral centroid (Sc)
Standard deviation (std_dev)	Geometric mean (mG)	Spectral S (Sspread)
Root mean square (rms)	Impulse factor (impInd)	Spectral skewness (Ssk)
Peak to rms (peak2rms)	Shape factor (shapeFa)	Spectral kurtosis (Sku)
Peak to peak (peak2peak)	Zero cross rate (zcrate)	Mean frequency (Mfreq)
Skewness (sk)	Lyapunov exponent (lyapExp)	Flatness (flat)
Kurtosis (ku)	Approximate entropy (apprEnt)	SpecEn (SpecEn)
3rd momentum (mom3)	Correlation dimension estimation	

**Table 3:** Dataset statistics.

No. Observations:	Dataset: 9902				Training Set (85%): 8416				Testing Set (15%): 1486			
Class:	D1	D2	D3	D4	D1	D2	D3	D4	D1	D2	D3	D4
No. Observations per Class:	2591	2437	2437	2437	2203	2071	2071	2071	388	366	366	366

**Table 4:** Statistical-based feature sets used in experiments.

Feature Set	Feature Type	No. of Features on One Axis	No. of Features on Three Axes
Fs69	statistical	X/Y/Z axis: 23	69
Fs72	statistical + paGMF	X/Y/Z axis: 24	72
Fs69_sel22	selected statistical	X axis: 7 Y axis: 8 Z axis: 7	22
Fs72_sel25	selected statistical + paGMF	X axis: 8 Y axis: 9 Z axis: 8	25

**Table 5:** Classification results for statistical-based representations of the vibration signals. Bold values represent the best classification results.

Classifier	Feature Set			
	*CI* (%) of the Mean Accuracy over 10 Runs			
	Fs69 *CI* (%)	Fs69_sel22 *CI* (%)	Fs72 *CI* (%)	Fs72_sel25 *CI* (%)
SVM—cubic	93.40 ± 0.367	**95.70 ± 0.346**	95.94 ± 0.416	**98.04 ± 0.176**
NN—wide	94.66 ± 0.354	**96.13 ± 0.291**	96.78 ± 0.352	**98.26 ± 0.207**

**Table 6:** Confusion matrices, one run of NN-wide feature sets: (**a**) Fs69_22sel and (**b**) Fs72_25sel.

(a) Fs69_22sel						(b) Fs72_25sel					
		Predicted class						Predicted class			
		D1	D2	D3	D4			D1	D2	D3	D4
True class	D1	375	1	2	10	True class	D1	383	2		3
	D2	4	362				D2	2	361	1	2
	D3	6	2	347	11		D3	1		362	3
	D4	12		11	343		D4	4	1	7	354
Precision (%)		94.46	99.18	96.39	94.23	Precision (%)		98.21	99.18	97.84	97.79
Recall (%)		96.65	98.91	94.81	93.72	Recall (%)		98.71	98.63	98.91	96.72
F1-score (%)		95.54	99.04	95.59	93.97	F1-score (%)		98.46	98.9	98.37	97.25
Accuracy = 96%						Accuracy = 98.25%					

**Table 7:** Feature sets based on harmonics and sidebands.

Feature Set	Feature Type	GMF Harmonics	First Pair of Sidebands	Second Pair of Sidebands	No. of Features on One Axis	No. of Features on Three Axes
BP_GMF	Band powers (BP)	6	-	-	6	18
BP_GMF_1sb		6	yes	-	6 × 3 = 18	54
BP_GMF_2sb		6	yes	yes	6 × 5 = 30	90
PA_GMF	Peak amplitudes (PA)	6	-	-	6	18
PA_GMF_1sb		6	yes	-	6 × 3 = 18	54
PA_GMF_2sb		6	yes	yes	6 × 5 = 30	90

**Table 8:** Accuracies of classifiers using GMF and sideband-based features. Bold values represent the best classification results.

Classifier	Feature Set *CI* (%) of the Mean Accuracy over 10 Runs					
	BP_GMF	BP_GMF_1sb	BP_GMF_2sb	PA_GMF	PA_GMF_1sb	PA_GMF_2sb
SVM-cubic	97.54 ± 0.236	99.39 ± 0.099	**99.73** ± 0.042	96.68 ± 0.180	99.3 ± 0.117	**99.72** ± 0.076
NN-wide	98.23 ± 0.124	99.49 ± 0.129	99.45 ± 0.141	97.45 ± 0.215	99.2 ± 0.170	99.29 ± 0.141

**Table 9:** Visual representation and classification in BP_GMF feature space.

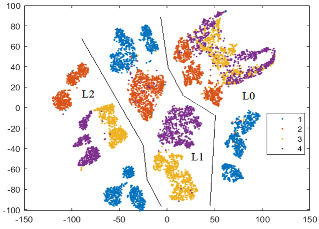			Predicted Class			
			D1	D2	D3	D4
	True class	D1	387	1		
		D2	3	361		2
		D3		3	354	9
		D4	1	7	10	348
	Precision (%)		98.98	97.04	97.25	96.94
	Recall (%)		99.74	98.63	96.72	95.08
	F1-score (%)		99.36	97.83	96.99	96.00
	Accuracy = 97.58%					
t-SNE projection in BP_GMF feature space	SVM-cubic, one run using BP_GMF features Confusion matrix and performance metrics					

**Table 10:** Visual representation and classification in the BP_GMF_1sb feature space.

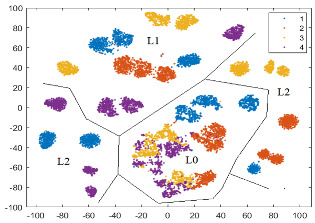			Predicted class			
			D1	D2	D3	D4
	True class	D1	387	1		
		D2	1	364		1
		D3			362	4
		D4			2	364
	Precision (%)		99.74	99.73	99.45	98.64
	Recall (%)		99.74	99.45	98.91	99.45
	F1-score (%)		99.74	99.59	99.18	99.05
	Accuracy = 99.39%					
t-SNE projection in BP_GMF_1sb feature space	SVM-cubic, one run using BP_GMF_1sb features Confusion matrix and performance metrics					

**Table 11:** Visual representation and classification in BP_GMF_2sb feature space.

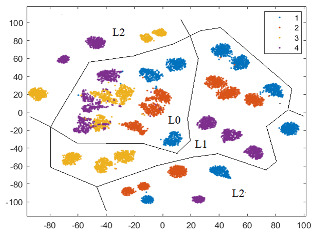			Predicted class			
			D1	D2	D3	D4
	True class	D1	388			
		D2		366		
		D3		1	364	1
		D4			2	364
	Precision (%)		100	99.73	99.45	99.73
	Recall (%)		100	100	99.45	99.45
	F1-score (%)		100	99.86	99.45	99.59
	Accuracy = 99.73%					
t-SNE projection in BP_GMF_2sb feature space	SVM-cubic, one run using BP_GMF_2sb features Confusion matrix and performance metrics					

**Table 12:** Accuracies achieved by several defect detection models applied to the same dataset.

Defect Detection Model	Feature Type	No. of Features on Three Axes	Classifier	Accuracy
GMF and sideband-based model	Band powers, six GMFs, and two sideband pairs	30 × 3 = 90	SVM-cubic	99.73%
2D-CNN-based model [31]	Extracted by 2D-CNN	576	Two hidden layers + softmax	99.63%
GMF and sideband-based model	Band powers, six GMFs, and one sideband pair	18 × 3 = 54	NN-wide	99.49%
GMF and sideband-based model	Band powers for six GMFs	6 × 3 = 18	NN-wide	98.23%
Statistical-based model	Selected statistical features + one harmonic peak amplitude	22 + 3 = 25	NN-wide	98.23%

## Data Availability

The experimental vibration data are available upon request.
